# A mutation that alters the 255th glutamate to lysine in RSKS-1/S6 kinase reliably extends the lifespan of *C. elegans*

**DOI:** 10.17912/micropub.biology.000322

**Published:** 2020-10-27

**Authors:** Jongsun Lee, Sieun S. Kim, Seung-Jae V. Lee

**Affiliations:** 1 Department of Biological Sciences, Korea Advanced Institute of Science and Technology, 291 Daehak-ro, Yuseong-gu, Daejeon, 34141, South Korea

**Figure 1:**
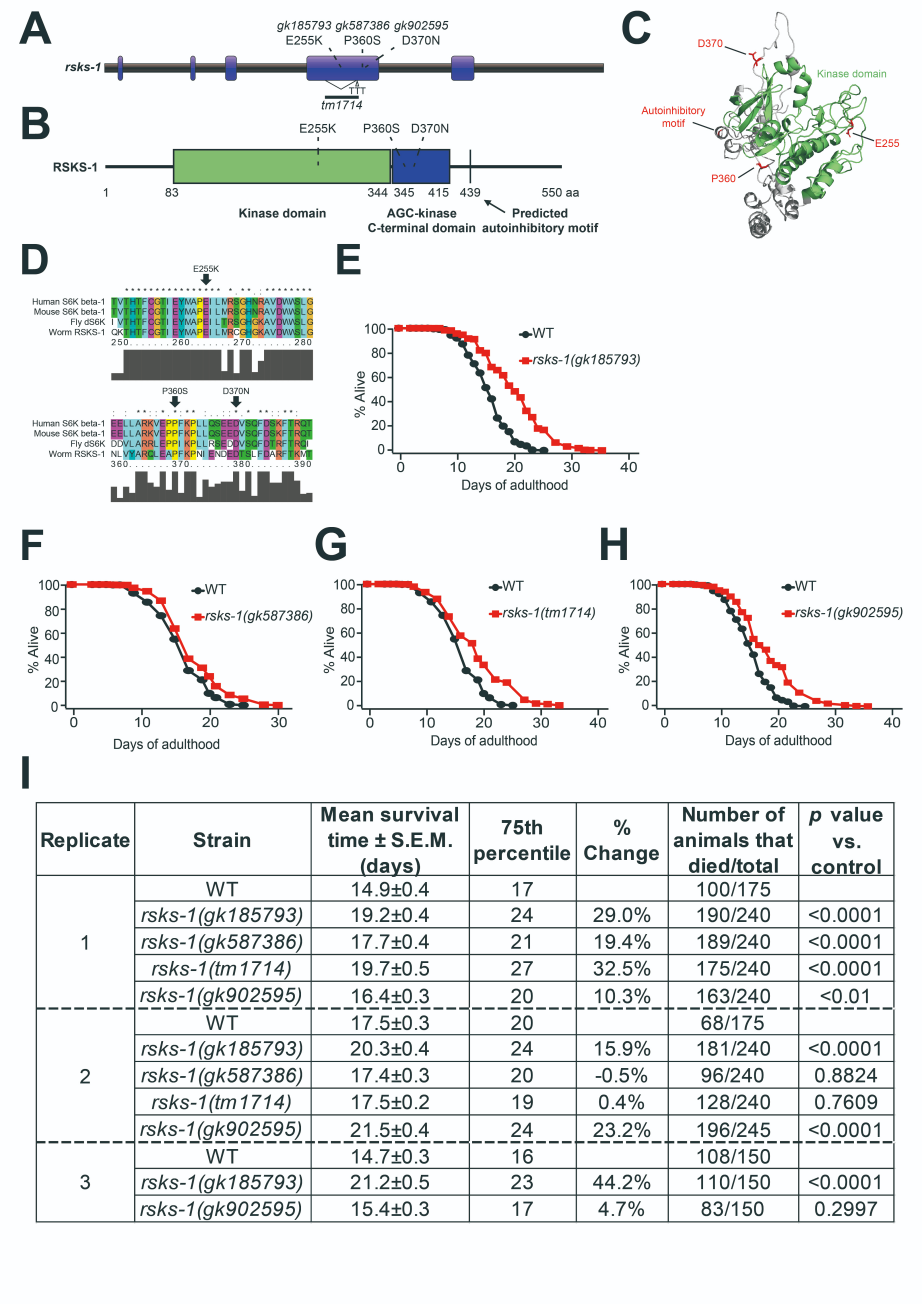
***rsks-1(gk185793)* that causes the E255K change reliably extends *C. elegans* lifespan at 20°C.** (**A**) A schematic showing the changes caused by *rsks-1* mutant alleles tested in this study. *gk185793*, *gk587386*, and *gk902595* cause mutations in the 4^th^ exon of *rsks-1* for resulting in E255K, P360S, and D370N changes, respectively. *tm1714* harbors a 484 base pair deletion and a three base pair (TTT) insertion at the 4^th^ exon of *rsks-1*. (**B**) The domain structure of RSKS-1/S6K protein in *C. elegans*. Amino acid (aa) numbers are shown at the bottom of the start and end residues of each domain. Kinase domain (83 aa – 344 aa), AGC-kinase C-terminal domain (345 aa – 415 aa), and predicted autoinhibitory motif (439 aa) are indicated. E255K is a point mutation in the kinase domain of RSKS-1. P360S and D370N mutations are in AGC-kinase C-terminal domain. (**C**) Three-dimensional structural modeling of worm RSKS-1. Mutation sites and the predicted autoinhibitory motif are painted as red. The E255 is located in the kinase domain (green), whereas P360 and D370 exist in the AGC-kinase C-terminal domain. (**D**) Alignment of amino acid sequences of human S6K, mouse S6K, *Drosophila melanogaster* (fly) dS6K, and *C. elegans* (worm) RSKS-1. A gray histogram represents the degree of amino acid conservation. Asterisks indicate residues that are completely conserved across species. Colons indicate any of the following amino acid residues that are strongly homologous: STA, NEQK, NHQK, NDEQ, QHRK, MILV, MILF, HY, and FYW. Periods indicate any of the following amino acid residues that are weakly homologous: CSA, ATV, SAG, STNK, STPA, SGND, SNDEQK, NDEQHK, NEQHRK, FVLIM, and HFY. (**E**–**H**) Pooled lifespan curves of *rsks-1(gk185793)* (**E**), *rsks-1(gk587386)* (**F**), *rsks-1(tm1714)* (**G**), and *rsks-1(gk902595)* (**H**) mutants compared with that of wild-type (WT: N2). (**I**) Statistical analysis of individual replicates of lifespan data. *p*-values were calculated using a log-rank (Mantel-Cox method) test. Biological replicates of the lifespan data were divided by dashed lines. S.E.M. represents the standard error of mean.

## Description

Mechanistic target of rapamycin (mTOR), a serine/threonine protein kinase, regulates biological processes in response to changes in nutrients or metabolites (Reviewed in Liu and Sabatini, 2020). Inhibition of mTOR signaling delays aging and increases lifespan in multiple organisms by downregulating substrates, including S6 kinase (S6K) that phosphorylates ribosomal subunit 6 (Reviewed in Kennedy and Lamming, 2016). Genetic inhibition of RSKS-1, the *Caenorhabditis elegans* S6 kinase, extends lifespan via decreasing protein synthesis and upregulating longevity-promoting factors (Reviewed in Johnson *et al.*, 2013; Lee *et al.* 2015). However, the longevity phenotypes of previously characterized loss-of-function *rsks-1* mutants are variable and greatly affected by environmental factors, such as temperatures (Seo *et al.*, 2013; Miller *et al.*, 2017). Here, we aimed at identifying *rsks-1* mutants whose lifespan phenotypes are robust and reproducible at 20°C, the standard temperature for *C. elegans* lifespan assays.

We measured the lifespan of three available *rsks-1* missense mutants generated by the Million Mutant Project (Thompson *et al.*, 2013) after outcrossing four times to wild-type (N2) strains, without treatment with 2′-deoxy-5-fluorouridine. The three missense mutant alleles are *rsks-1(gk185793)*, *rsks-1(gk587386)*, and *rsks-1(gk902595)*, which respectively cause E255 to K, P360 to S, and D370 to N changes (Fig. 1A–D). We also measured the lifespan of previously characterized *rsks-1(tm1714)* deletion mutants (Fig. 1A) (Chen *et al.*, 2013; Seo *et al.*, 2013). We found that *rsks-1(gk185793)*, which encodes RSKS-1 E255K, caused a consistent and strong longevity phenotype in all three independent trials (Fig. 1E and I). In contrast, *rsks-1(gk587386)* and *rsks-1(tm1714)* mutations extended lifespan in one out of two trials (Fig. 1F, G, and I). We found that *rsks-1(gk902595)* lengthened lifespan slightly (*p*<0.01) or robustly (*p*<0.0001), or did not (*p*=0.2997) in three trials (Fig. 1H and I). These variations in the lifespan of the three latter *rsks-1* mutants (Fig. 1F–I) may have been caused by small changes in environmental factors such as temperatures that affect the lifespan of *rsks-1* mutants (Seo *et al.*, 2013; Miller *et al.*, 2017). Together, our data indicate that *rsks-1(gk185793)* mutant allele that alters the acidic E255 residue to a basic K increases the lifespan of *C. elegans* reproducibly and robustly.

We speculate that the effects of *rsks-1(gk185793)* on lifespan were more robust than other missense alleles tested, because of following two reasons. First, E255 resides in the catalytic kinase domain of RSKS-1/S6K, but the others are located in the AGC-kinase C-terminal domain (Fig. 1A–D). Second, E to K, a change from an acidic to a basic amino acid, is likely to cause a bigger impact on the protein function than P to S and D to N changes. Future studies with additional missense mutations will clarify this issue.

Our study suggests that *rsks-1(gk185793)* mutant *C. elegans* is an excellent tool for aging research as a reliable genetic model for longevity conferred by reduced mTOR signaling. Additional future studies will enhance the value of *rsks-1(gk185793)* mutants for research on aging and mTOR signaling. First, further outcross of *rsks-1(gk185793)* mutants to wild-type worms to remove remaining background mutations will corroborate the causality of change in RSKS-1 for longevity. Alternatively or additionally, generation of the identical missense mutant allele by using CRISPR genome editing will be helpful. Second, confirming whether *rsks-1(gk185793)* is a reduction-of-function allele will be crucial for establishing the *rsks-1* mutant allele as a bona fide genetic model for reduced mTOR signaling. To this end, the assessment of phosphorylated ribosomal S6 and global protein synthesis levels will be required. Third, performing lifespan assays at various temperatures (e.g. 15°C and 25°C) and on different diets (e.g. *E. coli* HT115 and HB101 strains) may extend the utility of *rsks-1(gk185793)* mutants as a general longevity model. Lastly, E255 in *C. elegans* RSKS-1 is invariably conserved in mammalian S6K (Fig. 1D); therefore, it will be interesting to determine whether the orthologous E to K change in S6K extends lifespan in mice or is associated with human longevity.

## Methods

Amino acid sequence alignment of S6 kinase proteins in human, mouse, fly, and worm was conducted by using the ClustalX2 program (Thompson *et al.* 1997). The structural model of *C. elegans* RSKS-1 was generated with ModBase (Pieper *et al.* 2014) and subsequently visualized by using PyMOL program (The PyMOL Molecular Graphics System, Version 2.0, Schrödinger, LLC., https://pymol.org/2/).

Lifespan assays were performed as previously described (Park *et al.*, 2020). All lifespan measurements were performed from day 1 of adulthood. Animals were cultured at 20°C on nematode growth medium (NGM) agar plates seeded with *Escherichia coli* strain OP50. One hundred μl of OP50, which was cultured overnight at 37°C in liquid Luria broth (LB) media with 10 μg/ml streptomycin, was seeded on 35 mm NGM agar plates. Worms were synchronized at 20°C from hatching, and young adult worms were transferred to fresh OP50-seeded NGM plates. The worms were transferred again to fresh OP50-seeded plates every day until the worms stopped laying embryos. The deaths of worms were scored every two days. Worms that did not respond to a gentle touch with a sterilized platinum wire were determined as dead. Animals that burrowed, displayed internal hatching or vulval rupture, or crawled off the plates were censored but included in the statistical analysis.

Lifespan curves were generated and the statistical analysis was performed by using OASIS2 (https://sbi.postech.ac.kr/oasis2/) (Han *et al.*, 2016). *p*-values were calculated using a log-rank (Mantel-Cox method) test. The lifespan curves were pooled from two or three independent lifespan data obtained by two researchers.

## Reagents

*C. elegans* strains used in this study are as follows: wild-type Bristol N2, IJ1970 *rsks-1(gk185793)*, IJ2046 *rsks-1(gk587386)*, IJ109 *rsks-1(tm1714)*, and IJ1954 *rsks-1(gk902595)*. All mutant strains were outcrossed four times to Lee laboratory N2.
